# An Investigation into Unsafe Behaviors and Traffic Accidents Involving Unlicensed Drivers: A Perspective for Alignment Measurement

**DOI:** 10.3390/ijerph17186743

**Published:** 2020-09-16

**Authors:** Wafa Boulagouas, Susana García-Herrero, Rachid Chaib, Juan Diego Febres, Miguel Ángel Mariscal, Mébarek Djebabra

**Affiliations:** 1 Laboratory of Transportation Engineering and Environment, Department of Transportation Engineering, Faculty of Technology Sciences, University of Mentouri, Constantine 1, Constantine 25017, Algeria; wafa.boulagouas@umc.edu.dz (W.B.); rchaib@umc.edu.dz (R.C.); 2 Escuela Politécnica Superior, University of Burgos, 09006 Burgos, Spain; mariscal@ubu.es; 3 Department of Chemistry and Exact Sciences, Universidad Técnica Particular de Loja, Loja 110107, Ecuador; jdfebres@utpl.edu.ec; 4 Laboratory of Industrial Prevention Research, Health and Industrial Safety Institute, University of Batna 2, Batna 05078, Algeria; m.djebabra@univ-batna2.dz

**Keywords:** alignment, regulatory, behaviors, drivers, Bayesian, network, traffic, accidents, unlicensed, unsafe

## Abstract

Road traffic plays a vital role in countries’ economic growth and future development. However, traffic accidents are considered a major public health issue affecting humankind. Despite efforts by governments to improve traffic safety, the misalignment between the policy efforts and on-ground infringements, distractions and breaches reflect the regulatory failure. This paper uses the Bayesian network method to investigate unsafe behaviors and traffic accidents involving unlicensed drivers as a perspective for the regulatory alignment assessment. The findings suggest that: (1) unlicensed drivers are more likely to have unsafe driving behaviors; (2) the probability of being involved in a severe traffic accident increases when the drivers are unlicensed and decreases in the case of licensed drivers; (3) young drivers are noticeably more likely to engage in unsafe behaviors, usually leading to serious injuries and deaths, when their driving licenses are invalid; (4) women are more likely to engage in right-of-way violations and to have collisions with no serious injuries, contrary to unlicensed men drivers, who are involved in other types of traffic accidents resulting in serious injuries.

## 1. Introduction

Traffic safety has become a major public health concern all around the world [1,2,3]. The traffic safety problem is multidimensional, and many risk factors, i.e., technical factors (vehicles), environmental factors (the road and infrastructures), human factors (the road users) and their interactions, contribute to causing crashes [4,5,6]. Generally, two approaches to traffic safety studies have been established so far [7,8]. The first approach focuses on advancing engineering and enhancing traffic infrastructures, and the second approach is interested in the driver’s individual factors and driving behaviors. Indeed, these two approaches are complementary to each other within the systems perspective of Vision Zero [9]. This global vision is conditioned by the efficiency of the abovementioned approaches and involves a mix of initiatives to address safe mobility issues, i.e., vehicle safety, safety of infrastructures and promotion of road users’ behaviors [10,11].

In recent years, governments all around the world have undertaken several initiatives to enhance the traffic safety through [12,13]: (1) preventive measures in terms of education and information (i.e., communication campaigns and road safety advertisements); (2) corrective or sanctioning measures (i.e., coactive dissuasion and severe criminal penalties); (3) softer regulations based on economic sanctions (i.e., fines and insurance payments); and (4) license deprivation sanctions (i.e., suspension or withdrawal). Although the fundamental purpose of these regulations is to manipulate the conditions to change drivers’ behaviors and improve the results, the misalignment between policy efforts and breaches and on-ground traffic accidents reflects regulatory failure [14], hence the importance of the regulatory alignment assessment.

Regulatory alignment has firstly been used in the industrial workplace context and refers to the supportive attitudes, alignment and adherence to safety guidelines and policies to maintain an acceptable level of safety at the workplace [15,16]. To assess regulatory alignment, two main approaches have been adopted. The first approach, reactive in nature, investigates the outcomes of the implemented regulations (i.e., the number of accidents and fatalities), and the second approach, which is proactive, considers the assessment of the unsafe behaviors of the drivers, for instance, unlicensed driving, poor driving practices, disregarding the traffic signals and signs, and deliberate deviations from the recommended safe behaviors [17,18]. The first approach has been criticized due to its reactive nature and the fact that it cannot provide early warning information to empower authorities to take action prior to accidents. Thus, there has been a shift towards using leading indicators, such as unsafe behaviors, to more effectively measure the regulatory alignment and secure traffic safety goals [19,20].

Extensive studies have recognized the contribution of unsafe driving behaviors in traffic accidents and emphasized their overrepresentation in crashes, injuries and mortalities [21]. A review conducted to analyze 10 years of data (2005−2014) of fatal road crashes noted that unlicensed drivers were involved in 10% of fatal road crashes [22]. Likewise, an epidemiological study conducted in Japan on risk factors of fatality in traffic accidents found that the risk of fatal traffic accidents was higher among the unlicensed drivers [23]. It is noteworthy that when addressing unsafe driving behaviors, many research studies tended to examine the influence of additional risk factors (e.g., individual factors) as a way to characterize the traffic accidents. For instance, a study analyzing the changes in driving behaviors [24] found a significant relationship between the age and gender of the drivers and unsafe behaviors, i.e., violations, errors and lapses. Further, an analysis study of traffic accidents in China [25] asserted that males are overconfident, risk takers and less likely to respect traffic laws, and concluded that these drivers are at high risk of causing fatigue-related crashes.

Although much valuable knowledge has been obtained from the aforementioned studies, there is still little known about the relationship between unlicensed driving and other aberrant behaviors, on the one hand, and regulatory alignment and traffic accidents, on the other hand. To extend research beyond these trends, this paper conceptualizes the assessment of regulatory alignment of drivers considering the driving performance of unlicensed drivers.

There are good reasons to consider unlicensed driving an isolated risk behavior and to assess its moderating effect on the regulatory alignment. First, unlicensed driving negatively impacts the integrity of the driving management system [26]. That is to say, due to its illegal nature, it is difficult to estimate the driving performance of unlicensed drivers and their qualifications. Second, even though driving without a valid driving license is said to play an indirect causative role in traffic accidents, it is noteworthy that for many countries, it is still a serious problem, and many researchers [27,28,29] have suggested that there are behavioral differences between the aligned and the misaligned drivers that might be responsible for traffic accidents and their severe consequences. Finally, the purpose of the driving licensing system is identifying, monitoring and enforcing drivers’ behaviors using many programs and technologies (e.g., speed cameras) to facilitate the application of penalties and sanctions to traffic offenders. However, unlicensed driving is outside the licensing system, and it prevents authorities from tracking and managing unsafe behaviors [30].

This study investigates drivers’ unsafe behaviors and violations and the factors concerning traffic accidents considering driving license status and the influence of demographic variables (i.e., gender and age) as a way to better measure the regulatory alignment. Thus, it contributes to the literature in several ways. First, considering the growing concerns related to unlicensed driving and questions about the magnitude of traffic accidents involving unlicensed drivers, the present study examines their severity in depth. Second, whereas previous studies discussed unlicensed driving mainly as part of unsafe driving behaviors, this paper considers it as a separate risk behavior: it investigates the behaviors of the unlicensed drivers and elaborates the mechanism by which their behaviors are influenced by demographic factors so as to proactively measure the regulatory alignment and enable policymakers to set proper corrections. Finally, to overcome the shortcoming of the approaches used in road safety research and allow better estimation of the risk and uncertainties, the present paper uses the Bayesian network methodology to model the interplay between the driving license status and the behaviors of the drivers and therefore assess the impact of the influential factors, i.e., the demographics. For conceptual clarity, in this paper, the term “traffic accident” is used to refer to the road accidents involving motor vehicles and occurring on roads open to public circulation. The traffic accidents are studied based on two factors: the type (i.e., collision, run over and other types), and the severity of the outcomes (none/mild or severe injuries/death).

## 2. Materials and Methods

### 2.1. Bayesian Network

Road safety has been approached by many disciplines, for instance, transportation engineering, economics, social sciences, psychology and safety research, and each discipline examines a particular facet. Most studies deploy frequentist approaches, which are ad-hoc and account only for the expected values and do not carry the force of deductive logic [31]. As a response to the limitation of the frequentist methods, and to model the interplay between driving license status and drivers’ behaviors and to assess the impact of influential factors, the present study uses the Bayesian Network to derive posterior distributions from prior knowledge on the considered factors.

The deployment of the Bayesian methodology in recent decades has been developed for various subject areas for learning, modeling, forecasting and decision-making [32,33]. As regards the regulatory alignment and traffic safety research, Bayesian networks have been used to assess the safety impact of red-light cameras on the reduction of traffic signal violations [34], predict road safety hotspots [35], analyze the causation of road accidents [36,37], measure the influence the drivers’ behaviors and psychophysical factors on injury severity and distractions [38], measure the influence of the seat-belt use on the traffic accidents severity [39] and analyze the role of the journey purpose in road traffic injuries [40].

The Bayesian network is a formalism that combines graph and probability theory to provide a compact and natural representation offering effective inference and efficient learning [41]. The directed acyclic graph (DAG) represents the structure of the Bayesian network and qualifies the causal relationship between the variables of interest, while probability theory is responsible for the quantification of the network, that is, the quantification of the probabilistic causal relationships between the variables through the joint probability distribution (Equation (1)) based on the Bayes theorem (Equation (2)) [42]:

(1) $$P\left(X_{1},\;\ldots ,{\;X}_{n}\right)=\;\overset{n}{\underset{i=1}{\prod }}P(X_{i\;}|\mathrm{Parents}\;\left(X_{i}\right))$$

(2) $$P\left(A|B\right)=\;\frac{P\left(B|A\right)P\left(A\right)}{P\left(B\right)}$$

where P(X1, …, Xn) reflects the joint probability distribution, Parents (Xi) are parents of Xi, P(A|B) is the a posteriori probability, P(A) is the a priori probability and P(B|A) is the verisimilitude.

In this way, Bayesian networks consider the direct and conditional statistical dependencies between all of the study variables in one model. This flexibility allows the measurement of the influence of one or more variables on the target variable based on the a priori and a posteriori probabilities.

### 2.2. Validation Techniques

#### 2.2.1. Cross-Validation

The practicability of the obtained Bayesian network and its accuracy are assessed using the K-fold cross-validation approach, and the Bayes Net Toolbox [43,44] for Matlab [45] is deployed to perform the cross-validation, generate the Bayesian Network and compute the sensitivity analysis.

In this study, a 10-fold cross-validation has been considered. Accordingly, the data were divided into 10 folds, each containing 10% of the sample, and 90% of the sample was used to predict the sample in each of the corresponding folds. This operation was repeated ten times, and the entire sample prediction was obtained by joining the 10 folds. The evaluation of model skills was therefore measured using the area under the receiver operating characteristic (ROC) curve, called AUC. This standard measure for probabilistic and binary classifiers ranges between 0 and 1, where less than 0.5 corresponds to opposite and wrong predictions, 0.5 implies random prediction and non-reliable model and 1 refers to a perfect prediction and denotes that the model is reliable.

#### 2.2.2. Z-Test

To validate the conclusions driven from the sensitivity analysis, the statistical Z-test was used to measure the significance of the differences between the initial and a posteriori probabilities through the following hypotheses [46,47]:

(3) $$\left\{\begin{array}{c} H_{0}:\;P_{1}=\;P_{2}\\ H_{A}:\;P_{1}\neq \;P_{2} \end{array}\right\}$$

where P_1_ is the initial probability and P_2_ is the a posteriori probability.

Under the assumption of binomial distribution, the statistic test, Z_0_, is given by Equation (4):

(4) $${\;Z}_{0}=\;\frac{P_{1}-P_{2}}{\sqrt{\frac{P_{1}\;\left(1-P_{1}\right)}{n_{1}}+\;\frac{P_{2}\;\left(1-P_{2}\right)}{n_{2}}}}$$

where n_1_ and n_2_ are populations of the probabilities P_1_ and P_2_, respectively.

### 2.3. Data Acquisition

The dataset for the study was prepared from three years of official data (2016, 2017 and 2018) of traffic accidents in Spain. The original data were provided by the Traffic National Department of Spain and are made up of three databases: accidents, drivers and vehicles databases [48]:

- The drivers database contained data about the drivers involved in the accidents, for instance, age, gender and unsafe driving behaviors.
- The accidents database contained data about the type of accidents and the severity of the injuries, zone, etc.
- The vehicles database contained data about the vehicles involved in the accidents, for instance, the type of the vehicle, vehicle inspection and insurance.

In general, the three databases contained a total of 169 statistical elements (variables) collected from the “Form of Traffic Accidents with Victims” and 306,894 registered traffic accidents in which 524,785 drivers and 539,772 vehicles were involved. Each traffic accident has been registered with a unique registration ID; however, one or more driver(s)/vehicle(s) could have been involved in any registered traffic accident. The traffic accidents involving stationary vehicles, i.e., without drivers, were considered too.

**Figure 1:**
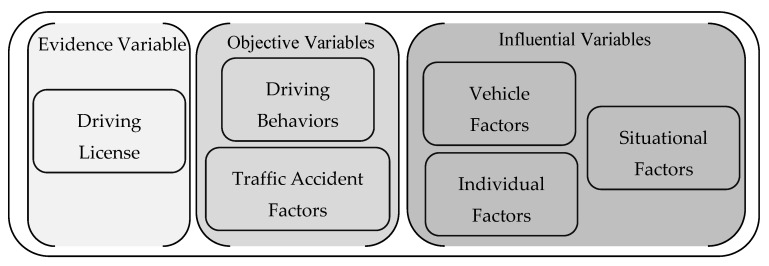
The variables of the sub-database used.

For the purpose of the present study, the dataset used was obtained by filtering the original drivers’ database to consider only car and motorcycle drivers and the study key variables, which were grouped into objective variables, i.e., the driving behaviors of the drivers and the traffic accident factors; the variables affecting the behaviors, i.e., the influential variables; and one evidence variable, i.e., the driving license (Figure 1). In doing so, the final dataset contains only a total of 467,431 drivers.

### 2.4. Study Variables

In the present study, special attention is paid to the unsafe behaviors of drivers based on the driving license status (Table 1). Unlicensed driving is defined as operating illegally motor vehicles on the road, putting these drivers themselves and other legitimate drivers at great risk [49]. In the context of the present study, the target variable is the driving license, including valid driving license and invalid driving license, which entails not only driving prior to the eligible age for licensing but also those unlicensed due to license expiration, suspension and cancellation, or inappropriate class of the license.

The objective variables are therefore the unsafe behaviors of drivers and traffic accident factors. In this study, unsafe behaviors were grouped into four main groups: distractive behaviors, speed infringement, other infringements and right-of-way violations (Table 2). As regards the traffic accident factors, two variables were considered, the type of the traffic accident—collision, run over and others—And the severity of the traffic accident, i.e., no-injury or mild injuries and serious injuries or death (Table 3).

The regulatory alignment in the context of road safety is a multidimensional construct and includes a wide range and multivariate combination of influencing factors, for instance, age, gender, decision-making behavior, personality, visibility, road type, zone, time and weather consideration and vehicle characteristics [50,51]. In the present study, the influential factors were grouped into three categories considering the available data: individual factors, situational factors and vehicle factors. However, the interplay between unlicensed driving and unsafe behaviors was assessed considering only the influence of the first group of factors, i.e., the individual factors, which include two demographic variables: age and gender of the drivers (Table 4).

## 3. Results

### 3.1. The Bayesian Network Validation

As explained in the methodology section, the validation of the obtained model was performed with a 10-fold cross-validation method and the results are given in Table 5.

All of the AUC scores range between 0.69 and 0.96 (with the exception of the affirmative status of the other infringements variable). These scores reflect the accuracy and high performance of the learned Bayesian network and confirm the practicability of the proposed approaches.

### 3.2. Z-Test

The differences between the probabilities used in the discussion of the sensitivity analysis results were examined using the statistical Z-test (results are given in Appendix A). The Z-test was conducted considering a confidence interval α of 95% (an admissible error of 5%) in a binomial distribution that proposes as limits +/−1.96 with Z_0.0/2_. To this end, all differences whose Z values are less than −1.96 or greater than 1.96 are acceptable statistical differences and significant.

### 3.3. Sensitivity Analysis

#### 3.3.1. Sensitivity Analysis of the Objective Variables Considering the Driving License Status

The initial probabilities for each of the objective variables considering the driving license status were computed, and results are given in Table 6 and Table 7. A confidence interval of 95% was considered to assess the statistical significance of the probabilities change.

The results in Table 6 show that the probabilities of the licensed drivers having safe driving behaviors are almost two times the probabilities of the unlicensed drivers.

For instance, the probability of committing no right-of-way violations when drivers are licensed is 50.84%, while the probability decreases to 23.27% when the drivers are unlicensed. Similarly, the probability of being involved in a minor traffic accident with no injuries is high at 90.79% when the drivers are licensed, and the probability decreases to 82.88% when the drivers have an invalid driving license.

However, according to Table 7, the probability of speeding is high when drivers are unlicensed, i.e., 12.38%, and decreases to 8.01% when the drivers have a valid driving license. In the case of right-of-way violations, the results show that licensed drivers have the highest probability, i.e., 35.08%, which decreases to 27.79% in the case of unlicensed drivers.

As regards the severity of traffic accidents, the probability of having a serious traffic accident leading to death is 9.21% when drivers are licensed and increases to 17.12% when the drivers have invalid driving licenses (a difference of 7.91%).

As far as the types of traffic accident, results show that the probability of having a collision is high in the case of licensed drivers, i.e., 77.65% and decreases to 73.56% in the case of unlicensed drivers; however, the probabilities of run-overs and other types of traffic accidents are high in the case of unlicensed drivers, i.e., 9.11% and 17.33% respectively.

According to these results, the status of the driving license is likely to have an important impact on the driving behaviors of drivers and the severity of traffic accidents.

#### 3.3.2. Sensitivity Analysis of the Objective Variables Considering the Driving License Status and the Individual Factors

According to the objective of the present paper and considering the learned Bayesian network that includes the joint probability distribution of the study variables, a sensitivity analysis was conducted to measure (1) the influence of individual factors and driving license status on drivers’ behaviors and (2) the influence of individual factors and driving license status on the type and severity of traffic accidents. A confidence interval of 95% has been considered to assess the statistical significance of probability change.

#### 3.3.3. Sensitivity Analysis of the Probabilities of the Drivers’ Behaviors Based on the Driving License Status and the Individual Factors

As regards the influence of individual factors, the sensitivity analysis results of Table 8 show that the probability of engaging in right-of-way violations increases from 27.79% (initial probability) in the case of the young unlicensed drivers (<25 years old) to 28.39% (a difference of 0.6%), and from 27.79% (initial probability) to 29.86% (a difference of 2.07%) in the case of older unlicensed drivers (>60 years old).

The probability of compliance with speed limits increases in the case of drivers older than 40 years old, regardless of the status of their driving licenses.

However, the probability decreases when drivers are younger than 25 years old from 67.03% (initial probability) to 60.34% (a difference of 6.69%) in case of valid driving licenses and from 31.80% (initial probability) to 26.98% (a difference of 4.82%) in the case of invalid driving licenses. Similarly, the results show that the probability of committing speed infringement increases in the case of the young licensed drivers by 6.47% and by 9.21% when they are unlicensed. However, in the case of the older drivers (>60 years old), the probability decreases regardless of the status of their driving licenses. The probability of not having other infringements increases in the case of the older licensed drivers (>60 years old) by 2.75% and decreases by 1.87% in the case of young licensed drivers (<25 years old).

For distracted driving behaviors, the sensitivity analysis results show that the probability of having no distracted driving decreases in the case of the young licensed drivers (<25 years old) by 2%. However, the probability increases by about 2% in the case of older licensed drivers (>60 years old).

Results of the sensitivity analysis of the influence of the gender variable on the behaviors of the drivers, considering the status of the driving license, are given in Table 9. In general, these results propose that the variable gender does not have an important influence on the driving behaviors of the drivers, regardless of the driving license status. However, some slight changes in the probabilities can be noticed. For instance, the probability of not engaging in right-of-way violations in the case of licensed men drivers shows an increase of 0.63%. However, the probability of engaging in these aberrant behaviors increases by about 2% in the case of women drivers regardless of the driving license status. For speed limit infringement, the probability increases by 0.48% in the case of the unlicensed men drivers.

#### 3.3.4. Sensitivity Analysis of the Probabilities of Traffic Accident Factors Based on Driving License Status and Individual Factors

Results of the sensitivity analysis of the influence of driving license status and individual factors on the traffic accidents are given in Table 10, Table 11, Table 12 and Table 13.

As regards the influence of the age variable and the driving license status on the type of the traffic accident, results of Table 10 show that the probability of being involved in a collision increases in the case of older drivers (>60 years old) by 2.25% in case of valid driving license and by 2.14% when their driving licenses are invalid. However, the probability of the younger drivers being involved in other types of traffic accidents increases by 3.06% when their driving licenses are valid and by 2.5% in the case of invalid driving licenses. As regards the severity of the traffic accidents, results in Table 11 show that in the case of older drivers (>60 years old), the probability of having a traffic accident with mild or no injuries increases regardless of the status of their driving licenses and it decreases in the case of serious traffic accidents.

However, with younger drivers (<25 years old), the probability of having a severe traffic accident increases, and does so more importantly when they are driving unlicensed, by 2%.

Results of the influence of drivers’ gender and driving license status on the probabilities of traffic accident types are summarized in Table 12.

These results reveal that the probability of having a collision increases by about 2% in the case of women drivers regardless of their driving licenses, while in the case of unlicensed men drivers, the probability increases in the case of other types of traffic accidents.

As regards the severity of the traffic accidents, results in Table 13 show that the probability of having a mild traffic accident with no injuries decreases in the case of men drivers regardless of their driving licenses; however, it increases in the case of women drivers by 2.79% when they have a valid driving license and, more importantly, when they hold an invalid driving license by about 7%. The same results show that the probability of having a serious traffic accident decreases in the case of women drivers regardless of their driving licenses. However, in the case of men drivers, the probability of having a serious traffic accident increases and, more importantly, when their driving licenses are invalid (an increase of 2.38%).

## 4. Discussion

Despite the improvements in the legislation and enforcement of laws targeting many traffic risk factors [52] and the fact that the drivers are aware of the adverse outcomes of engaging in unsafe driving behaviors, regulatory breaches continue to be witnessed and severe injuries, disabilities and deaths caused by traffic accidents continue to be recorded. Research studies have either shown the relationships between unsafe behaviors and traffic accidents or explained the contribution of unlicensed drivers to the frequency of traffic accidents. However, the relationship between these has not been investigated.

To assess the regulatory alignment, this study investigated the unsafe behaviors of unlicensed drivers. Such a focus first sheds light on the illegal driving of unlicensed drivers that escapes, in one way or another, follow-up strategies and road safety improvement projects. Second, such a focus proposes a proactive perspective for the assessment and monitoring of regulatory alignment, which is better than doing so reactively depending on traffic accident data, to help policymakers detect real deficiencies and make efficient and effective countermeasures.

In doing so, three years (2016, 2017 and 2018) of data were obtained from the Spanish National Traffic Department, and a Bayesian network has been deployed to provide predictions of changes in the probabilities and estimate how individual factors, i.e., demographic variables, impact the objective variables considering the statistical dependency relationships in the Bayesian network model.

This study demonstrated that licensed drivers are more likely to engage in safe driving behaviors such as respecting speed limits and less likely to be involved in run-over traffic accidents. In contrast, unlicensed drivers were found to engage in more unsafe behaviors like speeding and to have severe traffic accidents. This finding supports previous research studies [29,53] that have reported similar observations on risky driving behaviors of unlicensed drivers such as speeding and non-use of seatbelts, showing that unlicensed drivers form an important part of the profile of regulatory misalignment and that better traffic safety results could be achieved if policymakers and road safety authorities tackle unlicensed driving. As regards the severity of traffic accidents, results of the present study show that the probability of being involved in a minor traffic accident with no injuries is high when the drivers are licensed. In contrast, the probability of having a serious traffic accident leading to death increases when drivers have invalid driving licenses. This finding is in line with conclusions of many scholars [54,55], confirming that unlicensed drivers are more likely to be involved in fatal traffic accidents than licensed drivers, and the severity of such accidents is therefore high.

However, the present study marked some exceptions and found that the probability of licensed drivers engaging in right-of-way violations is higher than that of unlicensed drivers. In our opinion, the explanation lies in the complexity of the phenomenon of driving behavior, which, in such a particular case, is not exclusively influenced by the status of the driving license. The high probability of licensed drivers engaging in right-of-way violations could be explained by the fact that unlicensed drivers become “prudent drivers” in the streets because, in many countries, if the driver is cited for driving without a valid driving license, they may be fined, barred from obtaining a valid driving license for a period of time or incarcerated. Indeed, as reported by [56,57], drivers on roads or highways are more likely to be unlicensed than drivers on streets because on rural roads and highways, less public transport and taxi services are available and, considering the long distances, the likelihood of the unlicensed driver encountering the police is slim.

Another finding of notable interest is that both elder and younger drivers have unsafe driving behaviors. However, the results showed that each age group is likely to engage in some unsafe behavior more than others. For instance, young drivers are more likely to commit speed infringement, especially when their driving licenses are invalid. In contrast, older drivers (>60 years old) are more likely to engage in right-of-way violations. This finding supports the results of [57,58,59], confirming that young unlicensed drivers are the least committed to traffic instructions and violate traffic lights and use mobile phones the most. Adolescence is a critical developmental period that brings many important cognitive, social and emotional changes, affecting these young drivers’ ways of dealing with hazard and their proneness to engage in unsafe driving behaviors. Furthermore, as in many studies [60,61], the present study found that young drivers, and particularly young unlicensed drivers, are overrepresented in traffic accidents resulting in most of the serious injuries and deaths.

As regards the influence of the gender variable, the sensitivity results showed that women are more likely to engage in right-of-way violations and to have collisions. It was also found that the probability of having mild traffic accidents increases in the case of women unlicensed drivers. For men drivers, the results suggested that they are more likely to be involved in other types of traffic accidents and that the probability of having a serious traffic accident generally increases when their driving licenses are invalid. In general, these results are significantly consistent with many previous studies [62,63] that have agreed that women take fewer risks than men do when driving and are less involved in fatal traffic accidents.

To this end, it is clear that unlicensed driving is more than an unsafe behavior and that unlicensed driving motivates other disqualified driving performances. Thus, this study provides the most direct means for proactively estimating regulatory alignment and allows policymakers to better implement effective and efficient actions that might, first, buffer the impact of unlicensed driving unlicensed; second, reduce the likelihood of committing other unsafe behaviors; and finally, reduce the severity of traffic law violations and improve the alignment.

## 5. Conclusions

It is widely accepted that many people all around the world are killed or suffer disabilities due to traffic accidents. As a result, immense efforts are being made by road safety authorities all over the world to develop alternative ways to improve the behaviors of drivers at the wheel and therefore reduce the heavy costs of traffic accidents.

Relatively little previous research has investigated the mechanisms by which unlicensed driving affects driving performance and drivers’ regulatory alignment. In this paper, the interrelations between the alignment and compliance with traffic enforcement regulations, unlicensed driving, unsafe behaviors and traffic accidents were investigated.

As expected, findings of the present study confirmed that unlicensed driving exerts a significant negative impact on drivers’ behaviors and consequently their alignment with traffic regulations. Consequently, these findings provide evidence for promoting and improving traffic safety enforcements by targeting unlicensed driving in various safety education and enforcement programs.

### 5.1. Practical Implications

The present study provides a useful conceptualization of the regulatory alignment and the unsafe behaviors of unlicensed drivers that negatively affect traffic safety records. Accordingly, policymakers and practitioners could consider these results as the basis and empirical framework for interventions aimed at addressing unsafe behaviors and improving driving performance by paying more attention to the unlicensed driving problem. The interventions could fundamentally involve two important points: (i) the sanctions for the unlicensed driving should be reviewed, the laws tightened and special attention paid to unlicensed driving in prevention campaigns; and, (ii) since unlicensed driving is illegal and therefore goes underreported, moving towards using electronic driver licenses to deter unlicensed drivers from operating vehicles has become a necessity.

This study has also considered the use of big data techniques allowing, based on prior probabilities, the calculation of posterior probabilities, which is important to approach such public health problems and traffic safety studies.

### 5.2. Limitations and Future Research

The main limitation of the present study lies in the fact that the study variables were limited to those extracted from the database; however, there are many other unsafe driving behaviors and influential factors that could be of interest.

As regards the methodology, the machine-learning technique requires large amounts of data to train the data’s behavior; consequently, the concept of unlicensed drivers, in this paper, has grouped all of the categories. Thus, it is recommended that future research considers the influence of each category separately. This is because not all unlicensed drivers are similar. For example, a driver whose license was suspended or canceled due to a past driving offense is not the same as a driver whose license was expired. In considering each category separately, the interventions targeting unlicensed driving could be more specified and the focus could be directed to the disqualified drivers only. Moreover, this study has investigated only the influence of individual factors, and follow-up studies could investigate the influence of other factors.

## Figures and Tables

**Table 1:** Frequencies of the driving license status.

Driving License	Number of Cases			Total	Percentage	Comments
	2016	2017	2018			
Valid	85,321	84,284	83,701	25,3306	54.19%	Correct driving license
Invalid	2532	2139	2386	7057	1.51%	Inappropriate, expired, canceled, suspended, never had, total loss of points
Other	67,857	69,562	69,649	207,068	44.30%	The cases in which information about the driving license status are incomplete/not provided in the accident reports

**Table 2:** Frequencies of drivers’ behaviors.

Unsafe Behaviors		Number of Cases			Total	Percentage	Comments
		2016	2017	2018			
**Right-of-way violations**	No	50,530	48,676	48,417	147,623	31.58%	/
	Yes	32,597	33,666	34,117	100,380	21.47%	Non-respect of traffic signals, indications of a traffic agent or crosswalk, or similar reckless maneuvers, and others
	Unknown	72,583	73,643	73,202	219,428	46.94%	/
**Speed limit infringement**	No	65,579	64,692	62,393	192,664	41.22%	/
	Yes	8423	7674	7880	23,977	5.13%	Inadequate and excessive speed for road conditions or the established legal speed
	Unknown	81,708	83,619	85,463	250,790	53.65%	/
**Other infringements**	No	59,762	60,196	60,133	180,091	38.53%	/
	Yes	578	572	562	1712	0.37%	Driving without lights, dazzling, overload in the vehicle, open doors, excess occupants and similar
	Unknown	95,370	95,217	95,041	285,628	61.11%	/
**Distractions**	No	38,804	38,884	38,857	116,545	24.93%	/
	Technology-based distractions	326	371	354	1051	0.22%	Use of mobile phone, GPS, hands-free, radio, DVD and similar
	Other distractions	8767	9673	10,042	28,482	6.09%	smoking, interactions with occupants, thoughtful or abstracted, sleepy and similar
	Unknown	107,813	107,057	106,483	321,353	68.75%	/

**Table 3:** Frequencies of traffic accident factors.

Traffic Accident Factors	Number of Cases			Total	Percentage	Comments
	2016	2017	2018			
Traffic accident severity						
None/mild	142,783	142,946	143,530	429,259	91.86%	/
Serious injury/death	12,890	12,967	12,163	38,020	8.14%	Serious or fatal traffic accident
Total	155,673	155,913	155,693	467,279	100%	/
Traffic accident type						
Collision	119,137	116,680	115,177	350,994	75.11%	Frontal, side, multiple collision, collision by range or against obstacle
Run over	13,169	12,576	12,302	38,047	8.14%	Running over a person or an animal
Other	23,367	26,657	28,214	78,238	16.74%	/
Total	155,673	155,913	155,693	467,279	/	/

**Table 4:** Frequencies of the individual factors.

Influential Factors		Number of Cases			Total	Percentage
		2016	2017	2018		
**Individual factors**	**Age**					
	**<25**	21,541	20,962	20,248	62,751	13.42%
	25–40	60,012	58,462	57,984	176,458	37.75%
	41–60	54,369	55,976	57,696	16,8041	35.95%
	>60	16,604	17,393	17,627	51,624	11.04%
	Unknown	3184	3192	2181	8557	1.83%
	**Gender**					
	Men	110,443	111,068	111,061	332,572	71.15%
	Women	43,971	43,711	44,128	13,1810	28.20%
	Unknown	1296	1206	547	3049	0.65%

**Table 5:** The obtained AUC for the objective variables.

Objective Variables	Area Under the Curve (AUC)			
Right-of-way violations	No	Yes	Unknown	
	0.90	0.85	0.96	
Speed infringement	No	Yes	Unknown	
	0.94	0.82	0.95	
Other infringements	No	Yes	Unknown	
	0.94	0.53	0.94	
distractions	No	Technology-based distractions	Other distractions	Unknown
	0.91	0.69	0.85	0.91
Traffic accident type	Collision	Run-over	Other	
	0.69	0.75	0.77	
Traffic accident severity	None/mild	Serious injury/death		
	0.76	0.76		

**Table 6:** Probabilities of the safe behaviors of the drivers and traffic accident severity considering the driving license status.

Objective Variables		Valid Driving License	Invalid Driving License
**Safe behaviors**	No right-of-way violations	50.84% *	23.27% *
	No speed infringement	67.03% *	31.80% *
	No other infringements	63.09% *	34.96% *
	No distractions	43.49% *	18.26% *
**Traffic accident severity**	No injury or minor accident	90.79% *	82.88% *

Values highlighted with an asterisk, *, are statistically significant at a 95% confidence level.

**Table 7:** Probabilities of the unsafe behaviors of the drivers and traffic accident factors considering the driving license status.

Objective Variables			Valid Driving License	Invalid Driving License
**Unsafe behaviors**	Right-of-way violations		35.08% *	27.79% *
	Speed infringement		8.01% *	12.38% *
	Other infringements		0.60% *	0.39%
	Distractions	Technology-based distractions	0.37% *	0.44% *
		Other distractions	10.42% *	10.55% *
**Traffic accident factors**	Traffic accident severity	Serious injury or death	9.21% *	17.12% *
	Traffic accident type	Collision	77.65% *	73.56% *
		Run over	8.37% *	9.11% *
		Others	13.98% *	17.33%

Values highlighted with an asterisk, *, are statistically significant at a 95% confidence level.

**Table 8:** Probabilities for the behaviors of the drivers based on the driving license status and the age of the drivers.

Objective Variables		Initial Probabilities		Age	Driving License		
		Valid	Invalid		Valid	Invalid	Other
**Right-of-way violations**	No	50.84% *	23.27% *	<25	50.09% *	23.31% *	8.09% *
				25≤ Y ≤ 40	50.44% *	23.35% *	8.10% *
				40 < Y ≤ 60	51.31% *	23.73% *	8.62% *
				Y > 60	51.08% *	25.24% *	9.08% *
				Unknown	59.66% *	6.02% *	5.67% *
	Yes	35.08% *	27.79% *	<25	33.66% *	28.39% *	4.45% *
				25 ≤ Y ≤ 40	35.10% *	28.41% *	4.61% *
				40 < Y ≤ 60	35.26% *	27.54% *	4.73% *
				Y > 60	36.47% *	29.86% *	5.29% *
				Unknown	28.49% *	8.45% *	2.31% *
**Speed infringement**	No	67.03% *	31.80% *	<25	60.34% *	26.98% *	8.99% *
				25 ≤ Y ≤ 40	65.71% *	31.16% *	9.64% *
				40 < Y ≤ 60	69.26% *	34.02% *	10.59% *
				Y > 60	72.01% *	38.56%	11.69% *
				Unknown	66.50% *	8.15% *	5.94% *
	Yes	8.01% *	12.38% *	<25	14.48% *	21.59% *	2.36% *
				25 ≤ Y ≤ 40	8.69% *	13.43% *	1.44% *
				40 < Y ≤ 60	5.99% *	9.42% *	1.09% *
				Y > 60	4.81% *	7.81% *	0.88% *
				Unknown	4.84%	3.54% *	0.25% *
**Other infringements**	No	63.09% *	34.96% *	Y < 25	61.22% *	35.34% *	8.70% *
				25 ≤ Y ≤ 40	62.56% *	35.08% *	8.81% *
				40 < Y ≤ 60	63.84% *	35.43% *	9.39% *
				Y > 60	65.84% *	38.56%	10.47% *
				Unknown	39.20%	7.37% *	2.35% *
	Yes	0.60% *	0.39% *	Y < 25	0.61% *	0.44%	0.11% *
				25 ≤ Y ≤ 40	0.59% *	0.40%	0.11% *
				40 < Y ≤ 60	0.60% *	0.39%	0.11% *
				Y > 60	0.58% *	0.39%	0.11% *
				Unknown	0.48%	0.17%	0.05% *
**Distractions**	No	43.49% *	18.26% *	Y < 25	41.54% *	18.04% *	2.57% *
				25 ≤ Y ≤ 40	43.11% *	18.36% *	2.45% *
				40 < Y ≤ 60	43.81% *	18.49% *	2.49% *
				Y > 60	45.42% *	20.64% *	2.84% *
				Unknown	52.71% *	3.92% *	0.46% *
	Technology-based distractions	0.37% *	0.44% *	Y < 25	0.36% *	0.50%	0.03% *
				25 ≤ Y ≤ 40	0.37% *	0.45%	0.03% *
				40 < Y ≤ 60	0.37% *	0.43%	0.03% *
				Y > 60	0.39% *	0.47%	0.04% *
				Unknown	0.24%	0.10%	0.01% *
	Other distractions	10.42% *	10.55% *	Y < 25	10.40% *	10.85% *	0.71% *
				25 ≤ Y ≤ 40	10.41% *	10.75% *	0.67% *
				40 < Y ≤ 60	10.34% *	10.45% *	0.65% *
				Y > 60	10.91% *	11.57% *	0.72% *
				Unknown	6.35%	2.49% *	0.15% *

Values highlighted with an asterisk, *, are statistically significant at a 95% confidence level.

**Table 9:** Probabilities for the behaviors of the drivers based on the driving license status and the gender of the drivers.

Objective Variables		Initial Probabilities		Gender	Driving License		
		Valid	Invalid		Valid	Invalid	Other
**Right-of-way violations**	No	50.84% *	23.27% *	Men	51.47% *	23.70% *	8.33% *
				Women	49.37% *	23.16% *	8.27% *
				Unknown	54.24% *	7.74% *	6.83% *
	Yes	35.08% *	27.79% *	Men	34.24% *	27.69% *	4.57% *
				Women	37.03% *	29.58% *	4.82% *
				Unknown	32.49% *	10.63% *	3.30% *
**Speed infringement**	No	67.03% *	31.80% *	Men	67.01% *	31.93% *	9.98% *
				Women	67.07% *	33.16% *	10.06% *
				Unknown	67.60% *	10.52% *	7.68% *
	Yes	8.01% *	12.38% *	Men	8.05%*	12.86%*	1.35%*
				Women	7.91% *	11.43% *	1.36% *
				Unknown	6.47%	4.58%	0.63% *
**Other infringements**	No	63.09%*	34.96%*	Men	63.00% *	35.28% *	8.97% *
				Women	63.30% *	35.95% *	9.11% *
				Unknown	56.15% *	10.41% *	4.85% *
	Yes	0.60% *	0.39% *	Men	0.60% *	0.40%	0.11% *
				Women	0.58% *	0.39%	0.11% *
				Unknown	0.57%	0.18%	0.07% *
**Distractions**	No	43.49% *	18.26% *	Men	43.44% *	18.51% *	2.50% *
				Women	43.59% *	18.52% *	2.38% *
				Unknown	46.21% *	5.32% *	1.19% *
	Technology-based distractions	0.37% *	0.44% *	Men	0.37% *	0.45% *	0.03% *
				Women	0.38% *	0.46%	0.03% *
				Unknown	0.33%	0.14%	0.01% *
	Other distractions	10.42% *	10.55% *	Men	10.43% *	10.73% *	0.67% *
				Women	10.39% *	10.56% *	0.64% *
				Unknown	8.87%	3.29% *	0.34% *

Values highlighted with an asterisk, *, are statistically significant at a 95% confidence level.

**Table 10:** Probabilities of traffic accident types based on the driving license status and the age of the drivers.

Traffic Accident Type	Initial Probabilities		Age	Driving License		
	Valid	Invalid		Valid	Invalid	Other
**Collision**	77.65% *	73.56% *	Y < 25	75.26%	71.83% *	71.17% *
			25 ≤ Y ≤ 40	77.43% *	73.33% *	72.40% *
			40 < Y ≤ 60	78.01% *	73.87%	73.01% *
			Y > 60	79.90% *	75.70%	74.51% *
			Unknown	78.83% *	73.53%	74.37% *
**Run Over**	8.37% *	9.11% *	Y < 25	7.70% *	8.34%	6.80% *
			25 ≤ Y ≤ 40	8,32% *	8,97%	7,38% *
			40 < Y ≤ 60	8.58% *	9.31%	7.76% *
			Y > 60	8.57% *	9.66%	8.36%
			Unknown	10.60% *	10.78% *	9.64%*
**Others**	13.98% *	17.33%	Y < 25	17.04% *	19.83% *	22.03% *
			25 ≤ Y ≤ 40	14.25% *	17.70%	20.21% *
			40 < Y ≤ 60	13.41% *	16.82%	19.23% *
			Y > 60	11.53% *	14.65%	17.13% *
			Unknown	10.58% *	15.68%	15.99%

Values highlighted with an asterisk, *, are statistically significant at a 95% confidence level.

**Table 11:** Probabilities for the traffic accident severity based on the driving license status and the age of the drivers.

Traffic Accident Severity	Initial Probabilities		Age	Driving License		
	Valid	Invalid		Valid	Invalid	Other
**None/mild**	90.79% *	82.88% *	Y < 25	90.03% *	80.89% *	92.96% *
			25 ≤ Y ≤ 40	90.75% *	82.49% *	93.30% *
			40 < Y ≤ 60	90.90% *	83.23% *	93.52% *
			Y > 60	91.28% *	83.47% *	93.57% *
			Unknown	93.28%	93.62%	97.40% *
**Serious injury/death**	9.21% *	17.12% *	Y < 25	9.97% *	19.11% *	7.04% *
			25 ≤ Y ≤ 40	9.25% *	17.51% *	6.70% *
			40 < Y ≤ 60	9.10% *	16.77% *	6.48% *
			Y > 60	8.72% *	16.53% *	6.43% *
			Unknown	6.72%	6.38%	2.60% *

Values highlighted with an asterisk, *, are statistically significant at a 95% confidence level.

**Table 12:** Probabilities of the traffic accident types based on the driving license status and the gender of drivers.

Traffic Accident Type	Initial Probabilities		Gender	Driving License		
	Valid	Invalid		Valid	Invalid	Other
**Collision**	77.65% *	73.56% *	Men	76.88% *	73.05% *	72.30% *
			Women	79.43% *	75.20%	73.88% *
			Unknown	78.00%	74.11%	74.10%
**Run over**	8.37% *	9.11% *	Men	8.20% *	9.01% *	7.47% *
			Women	8.78% *	9.33%	7.93%
			Unknown	8.42%	10.41%	9.40% *
**Others**	13.98% *	17.33%		14.92% *	17.93% *	20.23% *
			Women	11.79% *	15.47%	18.19% *
			Unknown	13.58%	15.48%	16.49%

Values highlighted with an asterisk, *, are statistically significant at a 95% confidence level.

**Table 13:** Probabilities of traffic accident severity based on the driving license status and the gender of the drivers.

Traffic Accident Severity	Initial Probabilities		Gender	Driving License		
	Valid	Invalid		Valid	Invalid	Other
**None or mild**	90.79% *	82.88% *	Men	89.57% *	80.50% *	92.86% *
			Women	93.58% *	89.80% *	95.17% *
			Unknown	96.33% *	95.45% *	96.03% *
**Serious injury or death**	9.21% *	17.12% *	Men	10.43% *	19.50% *	7.14% *
			Women	6.42% *	10.20% *	4.83% *
			Unknown	3.67% *	4.55% *	3.97% *

Values highlighted with an asterisk, *, are statistically significant at a 95% confidence level.

## Appendix A

**Table A1:** Z-Test for safe behaviors and severity of traffic accidents considering driving license status.

Objective Variables		P_1_	P_2_	ΔP	Z
Safe behaviors of the drivers	No right-of-way violations	0.508	0.233	−0.276	161.826 **
	No speed infringement	0.670	0.318	−0.352	233.689 **
	No other infringements	0.631	0.305	−0.281	175.963 **
	No distractions	0.435	0.183	−0.252	137.042 **
Traffic accident severity	None/mild	0.908	0.829	−0.079	109.133 **

Z-Values highlighted with asterisks, **, are statistically significant differences at a 95% confidence level.

**Table A2:** Z-Test for unsafe behaviors and type and severity of traffic accidents considering driving license status.

Objective Variables		P_1_	P_2_	ΔP	Z
Unsafe behaviors of drivers	Right-of-way violations	0.351	0.278	−0.073	35.287 **
	Speed infringement	0.081	0.124	0.043	−15.496 **
	Other infringements	0.006	0.004	−0.002	0.876
	Technology-based distractions	0.004	0.004	0.001	−0.253
	Other distractions	0.104	0.106	0.001	−0.506
Traffic accident severity	Serious injuries or death	0.092	0.171	0.079	−32.479 **
Traffic accident type	Collision	0.777	0.736	−0.041	39.942 **
	Run over	0.084	0.091	0.007	−3.614 **
	Others	0.140	0.173	0.034	−18.253 **

Z-Values highlighted with asterisks, **, are statistically significant differences at a 95% confidence level.

**Table A3:** Z-Test for unsafe behaviors and type and severity of traffic accidents considering age and invalid driving license.

Objective Variables		Age	P_1_	P_2_	ΔP	Z
Unsafe behaviors of drivers	Right-of-way violations	<25	0.278	0.284	0.006	−3.132 **
		25 ≤ Y ≤ 40	0.278	0.284	0.006	−4.929 **
		40 < Y ≤ 60	0.278	0.275	−0.003	1.966 **
		Y > 60	0.278	0.299	0.021	−9.773 **
		Unknown	0.278	0.085	−0.193	62.847 **
	Speed infringement	<25	0.124	0.216	0.092	−53.807 **
		25 ≤ Y ≤ 40	0.124	0.134	0.011	−11.124 **
		40 < Y ≤ 60	0.124	0.094	−0.030	34.413 **
		Y > 60	0.124	0.078	−0.046	35.831 **
		Unknown	0.124	0.035	−0.088	43.019 **
	Other infringements	<25	0.004	0.004	0.001	−1.789
		25 ≤ Y ≤ 40	0.004	0.004	0.000	−0.569
		40 < Y ≤ 60	0.004	0.004	0.000	0
		Y > 60	0.004	0.004	0.000	0
		Unknown	0.004	0.002	−0.002	4.840 **
	Technology-based distractions	<25	0.004	0.005	0.001	−2.015 **
		25 ≤ Y ≤ 40	0.004	0.005	0.000	−0.536
		40 < Y ≤ 60	0.004	0.004	0.000	0.536
		Y >60	0.004	0.005	0.000	−0.949
		Unknown	0.004	0.001	−0.003	9.574 **
	Other distractions	<25	0.106	0.109	0.003	−2.272 **
		25 ≤ Y ≤ 40	0.106	0.108	0.002	−2.316 **
		40 < Y ≤ 60	0.106	0.105	−0.001	1.148
		Y > 60	0.106	0.116	0.010	−6.902 **
		Unknown	0.106	0.025	−0.081	46.232 **
Traffic accident severity	Serious injury or death	<25	0.171	0.191	0.020	−11.963 **
		25 ≤ Y ≤ 40	0.171	0.175	0.004	−3.682 **
		40 < Y ≤ 60	0.171	0.168	−0.004	3.286 **
		Y > 60	0.171	0.165	−0.006	3.420 **
		Unknown	0.171	0.064	−0.107	39.795 **
Traffic accident type	Collision	<25	0.736	0.718	−0.018	9.224 **
		25 ≤ Y ≤ 40	0.736	0.733	−0.003	2.106 **
		40 < Y ≤ 60	0.736	0.739	0.003	−2.239 **
		Y > 60	0.736	0.757	0.021	−10.578 **
		Unknown	0.736	0.735	−0.001	0.125
	Run over	<25	0.091	0.083	−0.008	6.519 **
		25 ≤ Y ≤ 40	0.091	0.090	−0.001	1.750
		40 < Y ≤ 60	0.091	0.093	0.002	−2.426 **
		Y > 60	0.091	0.097	0.006	−4.025 **
		Unknown	0.091	0.108	0.017	−4.942 **
	Others	<25	0.173	0.1983	0.025	−14.835 **
		25 ≤ Y ≤ 40	0.173	0.1770	0.004	−3.478 **
		40< Y ≤60	0.173	0.1682	−0.005	4.779 **
		Y > 60	0.173	0.147	−0.027	16.224 **
		Unknown	0.173	0.157	−0.017	4.157 **

Z-Values highlighted with asterisks, **, are statistically significant differences at a 95% confidence level.

**Table A4:** Z-Test for unsafe behaviors and type and severity of traffic accidents considering gender and invalid driving license.

Objective Variables		Gender	P_1_	P_2_	ΔP	Z
Unsafe behaviors of drivers	Right-of-way violations	Men	0.2779	0.2769	−0.001	0.985
		Women	0.2779	0.2958	0.018	−12.627 **
		Unknown	0.2779	0.1063	−0.172	30.532 **
	Speed infringement	Men	0.1238	0.1286	0.005	−6.363 **
		Women	0.1238	0.1143	−0.009	9.500 **
		Unknown	0.1238	0.0458	−0.078	20.438 **
	Other infringements	Men	0.0039	0.0040	0.000	−0.702
		Women	0.0039	0.0039	0.000	0.000
		Unknown	0.0039	0.0018	−0.002	2.717 **
	Technology-based distractions	Men	0.0044	0.0045	0.000	−0.662
		Women	0.0044	0.0046	0.000	−0.952
		Unknown	0.0044	0.0014	−0.003	4.386 **
	Other distractions	Men	0.1055	0.1073	0.002	−2.572 **
		Women	0.1055	0.1056	0.000	−0.104
		Unknown	0.1055	0.0329	−0.073	22.260 **
Traffic accident severity	Serious injury or death	Men	0.1712	0.1950	0.024	−27.025 **
		Women	0.1712	0.1020	−0.069	69.253 **
		Unknown	0.1712	0.0455	−0.126	32.957 **
Traffic accident type	Collision	Men	0.7359	0.7305	−0.005	5.379 **
		Women	0.7359	0.7520	0.016	−11.899 **
		Unknown	0.7359	0.7411	0.005	−0.653
	Run over	Men	0.0911	0.0901	−0.001	1.536
		Women	0.0911	0.0933	0.002	−2.431 **
		Unknown	0.0911	0.1041	0.013	−2.344 **
	Others	Men	0.1733	0.1793	0.006	−6.933 **
		Women	0.1733	0.1547	−0.019	16.322 **
		Unknown	0.1733	0.1548	−0.019	2.814 **

Z-Values highlighted with asterisks, **, are statistically significant differences at a 95% confidence level.

**Table A5:** Z-Test for unsafe behaviors and type and severity of traffic accidents considering age and valid driving license.

Objective Variables		Age	P_1_	P_2_	ΔP	Z
Unsafe behaviors of drivers	Right-of-way violations	<25	0.351	0.337	−0.014	7.060 **
		25 ≤ Y ≤ 40	0.351	0.351	0.000	−0.150
		40 < Y ≤ 60	0.351	0.353	0.002	−1.325
		Y > 60	0.351	0.365	0.014	−6.232 **
		Unknown	0.351	0.285	−0.066	13.370 **
	Speed infringement	<25	0.081	0.145	0.064	−43.688 **
		25 ≤ Y ≤ 40	0.081	0.087	0.006	−7.561 **
		40 < Y ≤ 60	0.081	0.060	−0.021	30.010 **
		Y > 60	0.081	0.048	−0.033	32.166 **
		Unknown	0.081	0.048	−0.033	13.848 **
	Other infringements	<25	0.006	0.006	0.000	−0.302
		25 ≤ Y ≤ 40	0.006	0.006	0.000	0.466
		40 < Y ≤ 60	0.006	0.006	0.000	0.000
		Y > 60	0.006	0.006	0.000	0.567
		Unknown	0.006	0.005	−0.001	1.588
	Technology-based distractions	<25	0.004	0.004	0.000	0.392
		25 ≤ Y ≤ 40	0.004	0.004	0.000	0.000
		40 < Y ≤ 60	0.004	0.004	0.000	0.000
		Y > 60	0.004	0.004	0.000	−0.694
		Unknown	0.004	0.002	−0.001	2.424 **
	Other distractions	<25	0.104	0.104	0.000	0.154
		25 ≤ Y ≤ 40	0.104	0.104	0.000	0.117
		40 < Y ≤ 60	0.104	0.103	−0.001	0.923
		Y > 60	0.104	0.109	0.005	−3.396 **
		Unknown	0.104	0.064	−0.041	15.222 **
Traffic accident severity	Serious injury or death	<25	0.092	0.100	0.008	−5.991 **
		25 ≤ Y ≤ 40	0.092	0.093	0.000	−0.494
		40 < Y ≤ 60	0.092	0.091	−0.001	1.343
		Y > 60	0.092	0.087	−0.005	3.735 **
		Unknown	0.092	0.067	−0.025	9.090 **
Traffic accident type	Collision	<25	0.774	0.753	−0.022	11.872 **
		25 ≤ Y ≤ 40	0.777	0.774	−0.002	1.885
		40 < Y ≤ 60	0.777	0.780	0.004	−3.051 **
		Y > 60	0.777	0.799	0.023	−12.057 **
		Unknown	0.777	0.788	0.012	−2.647 **
	Run over	<25	0.084	0.077	−0.007	5.884 **
		25 ≤ Y ≤ 40	0.084	0.083	−0.001	0.647
		40 < Y ≤ 60	0.084	0.086	0.002	−2.644 **
		Y > 60	0.084	0.086	0.002	−1.542
		Unknown	0.084	0.106	0.022	−6.652 **
	Others	<25	0.140	0.170	0.031	−19.314 **
		25 ≤ Y ≤ 40	0.140	0.143	0.003	−2.771 **
		40 < Y ≤ 60	0.140	0.134	−0.006	5.853 **
		Y > 60	0.140	0.115	−0.025	16.395 **
		Unknown	0.140	0.106	−0.034	10.108 **

Z-Values highlighted with asterisks, **, are statistically significant differences at a 95% confidence level.

**Table A6:** Z-Test for unsafe behaviors and type and severity of traffic accidents considering gender and valid driving license.

Objective Variables		Gender	P_1_	P_2_	ΔP	Z
Unsafe behaviors of drivers	Right-of-way violations	Men	0.351	0.342	−0.008	7.785 **
		Women	0.351	0.370	0.020	−12.982 **
		Unknown	0.351	0.325	−0.026	3.043 **
	Speed infringement	Men	0.081	0.081	−0.001	0.809
		Women	0.081	0.079	−0.002	2.252 **
		Unknown	0.081	0.065	−0.016	3.644 **
	Other infringements	Men	0.006	0.006	0.000	0.000
		Women	0.006	0.006	0.000	0.841
		Unknown	0.006	0.006	0.000	0.219
	Technology-based distractions	Men	0.004	0.004	0.000	0.000
		Women	0.004	0.004	0.000	−0.523
		Unknown	0.004	0.003	0.000	0.384
	Other distractions	Men	0.104	0.104	0.000	−0.144
		Women	0.104	0.104	0.000	0.315
		Unknown	0.104	0.089	−0.016	2.999 **
Traffic accident severity	Serious injury or death	Men	0.092	0.104	0.012	−17.992 **
		Women	0.092	0.064	−0.028	35.021 **
		Unknown	0.092	0.037	−0.055	16.145
Traffic accident type	Collision	Men	0.774	0.769	−0.005	5.771 **
		Women	0.777	0.794	0.018	−14.025 **
		Unknown	0.777	0.780	0.004	−0.465
	Run over	Men	0.084	0.082	−0.002	2.721 **
		Women	0.084	0.088	0.004	−4.667 **
		Unknown	0.084	0.084	0.001	−0.099
	Others	Men	0.140	0.149	0.009	−11.760 **
		Women	0.140	0.118	−0.022	21.410 **
		Unknown	0.140	0.136	−0.004	0.643

Z-Values highlighted with asterisks, **, are statistically significant differences at a 95% confidence level.

**Table A7:** Z-Test for safe behaviors and severity of traffic accidents considering age and invalid driving license.

Objective Variables		Age	P_1_	P_2_	ΔP	Z
Safe behaviors of drivers	No right-of-way violations	<25	0.233	0.232	−0.001	0.334
		25 ≤ Y ≤ 40	0.233	0.234	0.001	−0.677
		40 < Y ≤ 60	0.233	0.237	0.005	−3.808 **
		Y > 60	0.233	0.252	0.020	−9.805 **
		Unknown	0.233	0.060	−0.173	65.229 **
	No speed infringement	<25	0.318	0.270	−0.048	25.391 **
		25 ≤ Y ≤ 40	0.318	0.312	−0.006	4.938 **
		40 < Y ≤ 60	0.318	0.340	0.022	−16.548 **
		Y > 60	0.318	0.386	0.068	−30.072 **
		Unknown	0.318	0.082	−0.237	77.920 **
	No other infringements	<25	0.350	0.353	0.004	−1.870
		25 ≤ Y ≤ 40	0.350	0.351	0.001	−0.900
		40 < Y ≤ 60	0.350	0.354	0.005	−3.458 **
		Y > 60	0.350	0.386	0.036	−15.979 **
		Unknown	0.350	0.074	−0.276	94.831 **
	No distractions	<25	0.183	0.180	−0.002	1.345
		25 ≤ Y ≤ 40	0.183	0.184	0.001	−0.925
		40 < Y ≤ 60	0.183	0.185	0.002	−2.086 **
		Y > 60	0.183	0.206	0.024	−12.736 **
		Unknown	0.183	0.039	−0.143	66.000 **
Traffic accident severity	None/mild	<25	0.829	0.809	−0.020	11.963 **
		25 ≤ Y ≤ 40	0.829	0.825	−0.004	3.682 **
		40 < Y ≤ 60	0.829	0.832	0.004	−3.286 **
		Y > 60	0.829	0.835	0.006	−3.420 **
		Unknown	0.829	0.936	0.107	−39.795 **

Z-Values highlighted with asterisks, **, are statistically significant differences at a 95% confidence level.

**Table A8:** Z-Test for safe behaviors and severity of traffic accidents considering gender and invalid driving license.

Objective Variables		Gender	P_1_	P_2_	ΔP	Z
Safe behaviors of drivers	No right-of-way violations	Men	0.233	0.237	0.004	−4.469 **
		Women	0.233	0.232	−0.001	0.836
		Unknown	0.233	0.077	−0.155	31.832 **
	No speed infringement	Men	0.318	0.319	0.001	−1.230
		Women	0.318	0.332	0.014	−9.285 **
		Unknown	0.318	0.105	−0.213	38.014 **
	No other infringements	Men	0.350	0.353	0.003	−2.955 **
		Women	0.350	0.360	0.010	−6.625 **
		Unknown	0.350	0.104	−0.246	44.040 **
	No distractions	Men	0.183	0.185	0.002	−2.844 **
		Women	0.183	0.185	0.003	−2.149 **
		Unknown	0.183	0.053	−0.129	31.533 **
Traffic accident severity	None/mild	Men	0.829	0.805	−0.024	27.025 **
		Women	0.829	0.898	0.069	−69.253 **
		Unknown	0.829	0.955	0.126	−32.957 **

Z-Values highlighted with asterisks, **, are statistically significant differences at a 95% confidence level.

**Table A9:** Z-Test for safe behaviors and severity of traffic accidents considering age and valid driving license.

Objective Variables		Age	P_1_	P_2_	ΔP	Z
Safe behaviors of the drivers	No right-of-way violations	<25	0.508	0.501	−0.007	3.528 **
		25 ≤ Y ≤ 40	0.508	0.504	−0.004	2.863 **
		40 < Y ≤ 60	0.508	0.513	0.005	−3.306 **
		Y > 60	0.508	0.511	0.002	−1.035
		Unknown	0.508	0.597	0.088	−16.475 **
	No speed infringement	<25	0.670	0.603	−0.067	32.313 **
		25 ≤ Y ≤ 40	0.670	0.657	−0.013	9.979 **
		40 < Y ≤ 60	0.670	0.693	0.022	−16.907 **
		Y > 60	0.670	0.720	0.050	−23.803 **
		Unknown	0.670	0.665	−0.005	1.029
	No other infringements	<25	0.631	0.612	−0.019	9.037 **
		25 ≤ Y ≤ 40	0.631	0.626	−0.005	3.923 **
		40 < Y ≤ 60	0.631	0.638	0.007	−5.482 **
		Y > 60	0.631	0.658	0.028	−12.481 **
		Unknown	0.631	0.392	−0.239	44.868 **
	No distractions	<25	0.435	0.415	−0.020	9.301 **
		25 ≤ Y ≤ 40	0.435	0.431	−0.004	2.746 **
		40 < Y ≤ 60	0.435	0.438	0.003	−2.268 **
		Y > 60	0.435	0.454	0.019	−8.361 **
		Unknown	0.435	0.527	0.092	−16.931 **
Traffic accident severity	None/mild	<25	0.908	0.900	−0.008	5.991 **
		25 ≤ Y ≤ 40	0.908	0.908	0.000	0.494
		40 < Y ≤ 60	0.908	0.909	0.001	−1.343
		Y > 60	0.908	0.913	0.005	−3.735 **
		Unknown	0.908	0.933	0.025	−9.090 **

Z-Values highlighted with asterisks, **, are statistically significant differences at a 95% confidence level.

**Table A10:** Z-Test for safe behaviors and severity of traffic accidents considering gender and valid driving license.

Objective Variables		Gender	P_1_	P_2_	ΔP	Z
Safe behaviors of the drivers	No right-of-way violations	Men	0.508	0.515	0.006	−5.556 **
		Women	0.508	0.494	−0.015	9.428 **
		Unknown	0.508	0.542	0.034	−3.756 **
	No speed infringement	Men	0.670	0.670	0.000	0.188
		Women	0.670	0.671	0.000	−0.273
		Unknown	0.670	0.676	0.006	−0.670
	No other infringements	Men	0.631	0.630	−0.001	0.822
		Women	0.631	0.633	0.002	−1.397
		Unknown	0.631	0.562	−0.069	7.699 **
	No distractions	Men	0.435	0.434	−0.001	0.445
		Women	0.435	0.436	0.001	−0.647
		Unknown	0.435	0.462	0.027	−3.003 **
Traffic accident severity	None/mild	Men	0.908	0.896	−0.012	17.992 **
		Women	0.908	0.936	0.028	−35.021 **
		Unknown	0.908	0.963	0.055	−16.145 **

Z-Values highlighted with asterisks, **, are statistically significant differences at a 95% confidence level.
